# Polycyclic aromatic hydrocarbon exposure effects on trajectories of maternal and adolescent mental health

**DOI:** 10.1186/s13034-024-00804-1

**Published:** 2024-09-11

**Authors:** Mariah DeSerisy, Leilani Salas, Emiliya Akhundova, Dahiana Pena, Jacob W. Cohen, David Pagliaccio, Julie Herbstman, Virginia Rauh, Amy E. Margolis

**Affiliations:** 1 Department of Psychiatry, Vagelos College of Physicians and Surgeons, Columbia University, 1051 Riverside Drive, New York, NY 10032 USA; 2 Division of Child and Adolescent Psychiatry, New York State Psychiatric Institute, 1051 Riverside Drive, New York, NY 10032 USA; 3 Columbia College, Columbia University, 1130 Amsterdam Ave, New York, NY 10027 USA; 4 Department of Environmental Health Sciences, Mailman School of Public Health, Columbia University, 722 West 168th Street, New York, NY 10032 USA; 5 Columbia Center for Children’s Environmental Health, Mailman School of Public Health, Columbia University, 722 West 168th Street, New York, NY 10032 USA; 6 Heilbrunn Department of Population and Family Health, Mailman School of Public Health, Columbia University, 722 West 168th Street, New York, NY 10032 USA

**Keywords:** Maternal mental health, Child mental health, Polycyclic aromatic hydrocarbons, Intergenerational transmission

## Abstract

**Background:**

Parental psychological distress is a well-known risk factor for developmental psychopathology, with longer term parental distress associated with worse youth mental health. Neurotoxicant exposure during pregnancy is a risk factor for both poor maternal and youth mental health. The impact of one class of pollutant, polycyclic aromatic hydrocarbons (PAH), on long-term trajectories of maternal distress and youth self-reported mental health symptoms in adolescence has been understudied.

**Methods:**

PAH exposure was measured by DNA adducts in maternal blood sampled during the third trimester of pregnancy. Maternal distress, operationalized as maternal demoralization, was measured at 11 timepoints (prenatal to child age 16). Adolescent mental health symptoms were measured at age 13–15. Follow up analyses examined a subset of measures available at age 15–20 years. Structural equation modeling examined associations between PAH exposure during pregnancy and latent growth metrics of maternal distress, and between maternal distress (intercept and slope) and youth mental health symptoms in a prospective longitudinal birth cohort (*N* = 564 dyads).

**Results:**

Higher prenatal PAH exposure was associated with higher concurrent maternal distress. Prenatal maternal distress was associated with adolescent’s self-reported anxiety, depression, and externalizing problems. On average, maternal distress declined over time; a slower decline in mother’s distress across the course of the child’s life was associated with greater self-reported anxiety and externalizing problems in youth.

**Conclusions:**

Our findings are consistent with an intergenerational framework of environmental effects on mental health: PAH exposure during pregnancy affects maternal mental health, which in turn influences mental health outcomes for youth well into adolescence. Future research is necessary to elucidate the possible social and biological mechanisms (e.g., parenting, epigenetics) underlying the intergenerational transmission of the negative effects of pollution on mental health in caregiver-child dyads.

**Supplementary Information:**

The online version contains supplementary material available at 10.1186/s13034-024-00804-1.

## Background

Mounting evidence points to the deleterious impact of environmental chemical exposures, and especially pollution, on physical and mental health, particularly among vulnerable populations [1]. Polycyclic aromatic hydrocarbons (PAHs) are a class of carcinogenic pollutants generated from the incomplete combustion of fossil fuel, tobacco, and other organic materials [2]. Common sources of exposure to PAHs include air pollution and diet [3]. PAHs in air pollution are commonly associated with smoking, cooking, domestic heating, and the burning of incense and candles [3–9]. Dietary sources of PAHs largely depend on the method of cooking, preservation, and storage, with the highest levels present in charcoal-broiled or smoked meats, fats and oils, and some leafy vegetables and grains [3]. The EPA recommends that adult exposure to airborne PAHs remain less than 11.3 m^3^/day for women and 15.2 m^3^/day for men [10]; specific recommendations for exposure level in food products have not been established. Background levels of airborne PAHs measured in urban areas are reported to be between 0.15 and 19.3 ng/m^3^ in the United States [11] and dietary sources of PAHs are typically in the tens of micrograms per kilogram with maximum levels of 60 µg/kg (60,000 ng/kg). Together, these data indicate that exposure in the general adult population remains higher than government recommendations. Critically, children’s developing bodies and those of pregnant people are uniquely vulnerable to PAH effects, making high levels of PAH exposure particularly dangerous for youth and pregnant people [12–16]. Further, marginalized populations experience even greater levels of exposure than the general population as a result of environmental injustice and associated structural factors [17]. Nevertheless these populations remain understudied but experience high levels of mental health problems.

PAHs are known to cause deleterious physical and mental health effects, including cancer and disruptions to gene, reproduction, and immune system function (for review: [18]). Meta-analytic evidence indicates that concurrent PAH exposure in adults is associated with elevated symptoms of depression [19–21]; animal and human cell models suggest that that this may operate through inflammatory processes associated with PAH exposure [22–24]. Importantly, pregnancy represents a unique and critical period of vulnerability during the adult life course [25] and exposure to PAHs during this period may heighten effects of PAH exposure. Thus, PAHs are a viable but unstudied risk factor for maternal mental health symptoms in the perinatal or postpartum periods. However, no studies have yet examined the effects of PAH exposure during pregnancy on mental health symptoms in pregnant people.

In youth, meta-analytic evidence indicates that prenatal exposure to PAH is associated with increased risk for numerous neurodevelopmental problems, including problems with social behavior, attention, and motor skills [21]. Prenatal PAH exposure has also been linked to anxiety and depression symptoms in youth, though meta-analytic support for these findings are mixed [21, 26]. Evidence from both animal and human studies suggest that prenatal exposure to PAH in air pollution may alter the trajectory of fetal brain development [27–29], potentially through inflammatory processes, leading to cascading changes in neural structure and function [29, 30] and increased vulnerability to later chemical or social exposures [31]. Given that effects of prenatal PAH exposure on children’s mental health have been previously examined, the current study aims to elucidate the effects of PAH exposure during pregnancy on maternal mental health in order to better understand the indirect pathway through which PAH exposure during pregnancy may increase risk for long term mental health problems in youth.

Maternal psychological distress is a robust and well-replicated risk factor for child psychopathology and mental health symptoms [32–34]. The mechanisms driving associations between maternal psychological distress and child mental health symptoms are diverse and intersecting, as has been described in several theoretical frameworks. For example, the family stress model posits that maternal psychological distress can influence parenting style [35], early attachment and bonding [36], and family processes, such as stress, cooperative caregiving, and home environment [37]. Theories of intergenerational psychiatry posit that transmission of mental health symptoms may also be biological, with emerging evidence suggesting genetic, epigenetic, and physiological (e.g., oxytocin, immune, etc.) mechanisms underlying offspring vulnerability to psychiatric disorders [38–40]. Together, these frameworks highlight the importance of understanding, and potentially intervening on, risk factors for maternal psychological distress given its association with poor child mental health outcomes [41].

Psychological distress encompasses a range of mental health symptoms from diagnosable disorders to relatively transient, minor stress [42]. Experiences of incompetence in the face of distress are described as demoralization [43, 44]. Importantly, demoralization is related to, but distinct from, depression, and elevated demoralization has been linked to risk for depression and suicidality beyond symptoms of depression alone [45, 46].

Demoralization is operationalized as both subjective psychological distress (e.g., feeling bothered by sadness, restlessness, or fear) and the inability to cope with those distressing feelings (e.g., feeling helpless, lonely, or unsupported). Demoralization in adults is associated with elevated risk for psychiatric disorders as well as symptoms of depression and anxiety (for review: [47]). Demoralization in mothers has been linked to numerous physical and mental health outcomes in children, including upper respiratory distress, poor immune function, anxiety, depression, and attention problems (e.g., [26, 48–51]). Notably, however, maternal mental health sequalae related to mothers’ own demoralization have not yet been investigated.

Risk for maternal demoralization is not well understood. Existing, though limited, research has focused on psychosocial factors associated with maternal demoralization, such as unemployment, low educational achievement, marital conflict, dependence on public assistance, single status, and low income [52–56]. As described above, environmental chemical exposures, particularly during the highly vulnerable pregnancy period, may also increase risk for demoralization. Although associations between PAH and demoralization have not been examined, associations between PAHs and adult depression appear to be strongest in women [31, 53–55]. Evidence from animal models indicates potential biologic pathways through which air pollution exposure during gestation results in demoralization-like behaviors in both dams and offspring [31, 57–59]. For example, dams exposed to air pollution demonstrated fewer caregiving behaviors when compared to non-exposed dams [59]; offspring of dams exposed to air pollutants during gestation demonstrated behavioral markers of demoralization (e.g., floating during forced swim; immobility during tail suspension) [31, 57, 58]. Further research is needed to understand the psychosocial and biological pathways through which PAH-related changes in maternal behavior is associated with offspring outcomes.

The current study is focused on understanding one possible indirect pathway through which exposure to PAH during pregnancy could drive maternal demoralization and in turn increase adolescents’ mental health problems. We hypothesize that maternal exposure to PAH during pregnancy will increase risk for demoralization in mothers prenatally and over time. Furthermore, we hypothesize that PAH-related increases in maternal demoralization over childhood will be associated with higher self-reported anxiety, depression, and externalizing problems as reported by their adolescent children.

## Methods

### Participants

Detailed demographic and recruitment information regarding the Columbia Center for Children’s Environmental Health (CCCEH) Mothers and Newborns prospective birth cohort have been previously published [60]. Briefly, Black and Latiné women who resided in Washington Heights, Harlem, or the South Bronx in New York City were recruited between 1998 and 2006 through local prenatal care clinics. Recruited women were non-users of tobacco products or illicit drugs, between the ages of 18 and 35 years, and free of diabetes, hypertension, or known HIV, and had initiated prenatal care by the 20th week of pregnancy. The full cohort included data from 727 mother-child dyads. Of the 727 dyads enrolled in the Mothers and Newborns cohort, 440 had available data for all predictors of interest (i.e., PAH-deoxyribonucleic acid (DNA) adducts, maternal demoralization, and baby’s sex at birth) and were included in the current analyses.

### Measures

#### Maternal demoralization

Maternal demoralization was measured using the Psychiatric Epidemiology Research Instrument–Demoralization (PERI-D; [61]), a 27-item scale measuring eight composite domains of non-specific psychological distress (perceived physical health, sadness, poor self-esteem, dread, anxiety, confused thinking, hopelessness/helplessness, and psychophysiological symptoms). Previous studies examining the trajectories of maternal mental health on child behavior outcomes have primarily focused on the early childhood period (e.g., 62–65) and only one has examined maternal mental health into adolescence and young adulthood [66]. Maternal demoralization was collected at 11 timepoints between mother’s third trimester of pregnancy and child age 16 (Prenatal, 6 Months, 12 Months, 24 Months, 36 Months, 5 Years, 7 Years, 9 Years, 11 Years, 14 Years, 16 Years; See Table 1). Total sum score was used in all analyses.

**Table 1:** Demographic characteristics of participants at each study visit

	Prenatal	6 Months	12 Months	24 Months	36 Months	5 Year	7 Year	9 Year	11 Year	14 Year	16 Year
N	530	502	509	484	489	510	491	454	366	351	260
Average maternal demoralization (SD)	30.7 (17.13)	30.52 (18.80)	28.38 (17.11)	28.68 (17.33)	28.38 (17.11)	27.23 (18.14)	26.25 (18.75)	25.46 (17.87)	26.01 (17.88)	28.45 (19.36)	28.54 (17.92)
Sex (% male)	256 (48.30)	242 (48.21)	245 (48.23)	225 (46.58)	235 (48.16)	242 (47.54)	235 (47.96)	213 (47.02)	168 (45.90)	157 (44.86)	117 (45.0)
Maternal years of education at prenatal visit (SD)	11.89 (2.20)	11.92 (2.16)	11.88 (2.19)	11.94 (2.13)	11.9 (2.19)	11.9 (2.17)	11.86 (2.15)	11.87 (2.13)	11.94 (1.95)	11.82 (2.16)	11.87 (2.06)
Child gestational age (SD)	39.20 (1.29)	39.2 (1.30)	39.22 (1.28)	39.18 (1.31)	39.22 (1.27)	39.21 (1.28)	39.22 (1.28)	39.24 (1.26)	39.22 (1.30)	39.21 (1.31)	39.23 (1.37)
PAH-exposed (% yes)	171 (41.71)	157 (40.26)	162 (41.12)	150 (40.11)	150 (39.27)	161 (40.66)	157 (40.67)	148 (39.89)	115 (36.86)	112 (38.23)	85 (38.29)
Heat season (% yes)	267 (50.66)	252 (50.50)	254 (50.20)	240 (49.90)	241 (49.59)	254 (50.10)	238 (48.77)	220 (48.78)	173 (47.53)	166 (47.43)	123 (47.49)
Smoker at home (% yes)	175 (33.33)	171 (34.41)	166 (32.94)	162 (33.82)	161 (33.26)	164 (32.48)	159 (32.72)	145 (32.29)	124 (34.25)	121 (34.87)	83 (32.30)
Maternal IQ (SD)	85.21 (13.30)	85.1 (13.33)	84.88 (12.90)	85.02 (13.24)	85.05 (13.36)	84.99 (13.20)	85.2 (13.20)	84.95 (12.78)	84.89 (13.04)	849 (12.99)	84.24 (12.50)
Latine	334	318	327	301	304	315	301	272	227	211	167
Black	196	184	182	183	185	195	190	182	139	140	93

PAH polycyclic aromatic hydrocarbons, SD standard deviation

#### Adolescent socioemotional functioning

Previous studies of associations between maternal mental health symptoms and child behavior have relied on parent-report of children’s symptomatology (e.g., [62–66]. However, when compared to self- or teacher-reports of children’s behaviors, mothers experiencing elevated levels of psychological distress are known to overreport mental health symptoms for their children, particularly related to externalizing behavior problems [67–70]. Furthermore, adolescents tend to be better reporters of their own mental health symptoms [71]. Therefore, we extend previous literature and examine adolescents’ self-report of their anxiety, depression, and externalizing problems at age 13–15. The Revised Children’s Manifest Anxiety Scale (anxiety symptoms) and Youth Self-Report (externalizing behavior problems) were repeated between adolescent ages 15–20. All symptom scales were z-scaled prior to inclusion in the models. Participants with z-scale values above or below 3 standard deviations were winsorized to the next non-outlier value.

*The Revised Children’s Manifest Anxiety Scale* (RCMAS; [72–74] is a 37-item measure of youth anxiety symptoms. Items are rated on a 0–1, presence or absence scale. Total scores are generated by summing across 28 anxiety items, ignoring the 9 social desirability items. Scores range from 0 to 28. Total scores above 19 may indicate clinically significant anxiety [75].

*The Center for Epidemiologic Studies Depression Scale* (CES-D; [76] is a 20-item self-report measure of depression symptoms. Items are rated on a 0–3 scale indicating the frequency of experience (e.g., rarely or none of the time to most or all of the time). Total scores are generated by reverse scoring positively worded items and then summing across all items. Scores range from 0 to 60, with scores above 15 indicating significant levels of depression symptoms [77].

*The Youth Self-Report of the Children’s Behavior Checklist* (YSR; [78]) is a 112-item self-report measure of socioemotional functioning. The YSR derives several subscales, of which the Externalizing Problems subscale was used in the current analyses. The Externalizing Problems subscale contains items reflecting rule-breaking and aggressive behavior, such as “I destroy things belonging to others” and “I disobey my parents.” Items are rated 0–2, not true to very true or often true. Subscale scores are generated by summing across 34 relevant items and converting to t-scores based on youth age and sex. T-scores between 60 and 64 indicate risk for problem behaviors; t-score ≥ 65 indicate clinically significant externalizing problems.

### PAH exposure

PAH exposure was measured by DNA adducts in maternal blood sampled during the third trimester of pregnancy. DNA adducts integrate PAH exposure over a period of months (estimated half-life: 3–4 months in blood [79, 80]) and so represent individual variation in exposure, absorption, metabolic activation, and DNA repair. Thus, DNA adducts provide a biological dosimeter incorporating both exposure and biological susceptibility. Adducts reflect multiple biological mechanisms for the pathogenic effects of PAH, including DNA damage, detoxification, and DNA repair [81]. Further, they may also play a role in downstream epigenetic alterations underlying individual variation in behavior [82–84]. The assay leveraged in the current study specifically measures adducts formed by Benzo[a] Pyrene as a proxy for PAH-DNA because it is considered a representative PAH and is highly correlated with other PAHs [60]. The method used to measure the adducts has been described in detail in prior work [60]. In brief, a total of 100 ug DNA was dissolved in 0.1 N HCL and acid hydrolysis was completed at 90 °C for 6 h. The resulting solution was analyzed in a Shimadzu HPLC system with an automatic sample injector and RF-10Axl spectrofluorometric detector. The tetrol concentrations were calculated by comparing the areas of samples to be analyzed with an external calibration curve, generated from the fluorescence peak of an authentic BPDE tetrol standard, every time a set of samples was analyzed. Calibration was conducted using DNA from calf thymus, alone (background) and added to 2, 4, and 8 pg anti-BPDE tetrol. These standard solutions were then treated in the same way as the tested samples (hydrolyzed in 0.1 N HCl at 90 °C for 6 h). The minimum correlation coefficient was 0.98 and the mean coefficient of variation for analyses repeated on different days was 12%. This assay can measure 0.25 adducts/10 − 8 nucleotides. PAH exposure (DNA adducts) was used as a dichotomous variable (detectable v non-detectable). 40% of the mothers in the sample had DNA adduct levels in the detectable range.

### Analytic plan

We estimated a latent growth curve model of maternal demoralization over a 16 year period beginning in the 3rd trimester of pregnancy using the *growth* function of lavaan package in R studio version 2023.06.1 [85, 86]. Of note, latent growth curve models are distinct from latent class models in that they use the continuous effects of all predictors on the continuous, data-derived outcomes, namely the intercept and slope scores. Intervals between slope loadings were scaled to reflect the intervals (i.e., months) between study visits. Full information maximum likelihood estimation was used to address missing demoralization data. Primary analyses included mothers with any available demoralization data. Sensitivity analyses included only mothers with demoralization data at more than 3 time points (*N* = 548). To control for potential confounding, the following covariates known to be associated with PAH exposure and maternal mental health outcomes were included in the model: maternal years of education as a proxy for socioeconomic status, child’s gestational age, mother’s age at birth, maternal intelligence (Test of Nonverbal Intelligence (TONI); [87]), maternal ethnoracial background (Black/Latiné), mother’s nativity (US born/emigrated), the presence of a smoker in the home, and heating season when PAH was measured (See supplementary methods for details). Gestational age, years of education, and maternal intelligence were z-scaled. Model fit was assessed using comparative fit index (CFI), root mean square error of approximation (RMSEA) and the standardized root mean square residual (SRMR), as these are recommended for smaller sample sizes (*N* < 500 [88, 89]). The RMSEA should be close to zero with a significance value > 0.05 [90, 91], CFI > 0.90 [92, 93], SRMR < 0.08 [94]). Nonlinear trends in maternal demoralization trajectories were explored using freely estimated lambda parameters in sensitivity analyses. Such parameters allow for the coefficients of the change in demoralization over time to be estimated from the data, rather than using fixed linear effects [95].

**Fig. 1:**
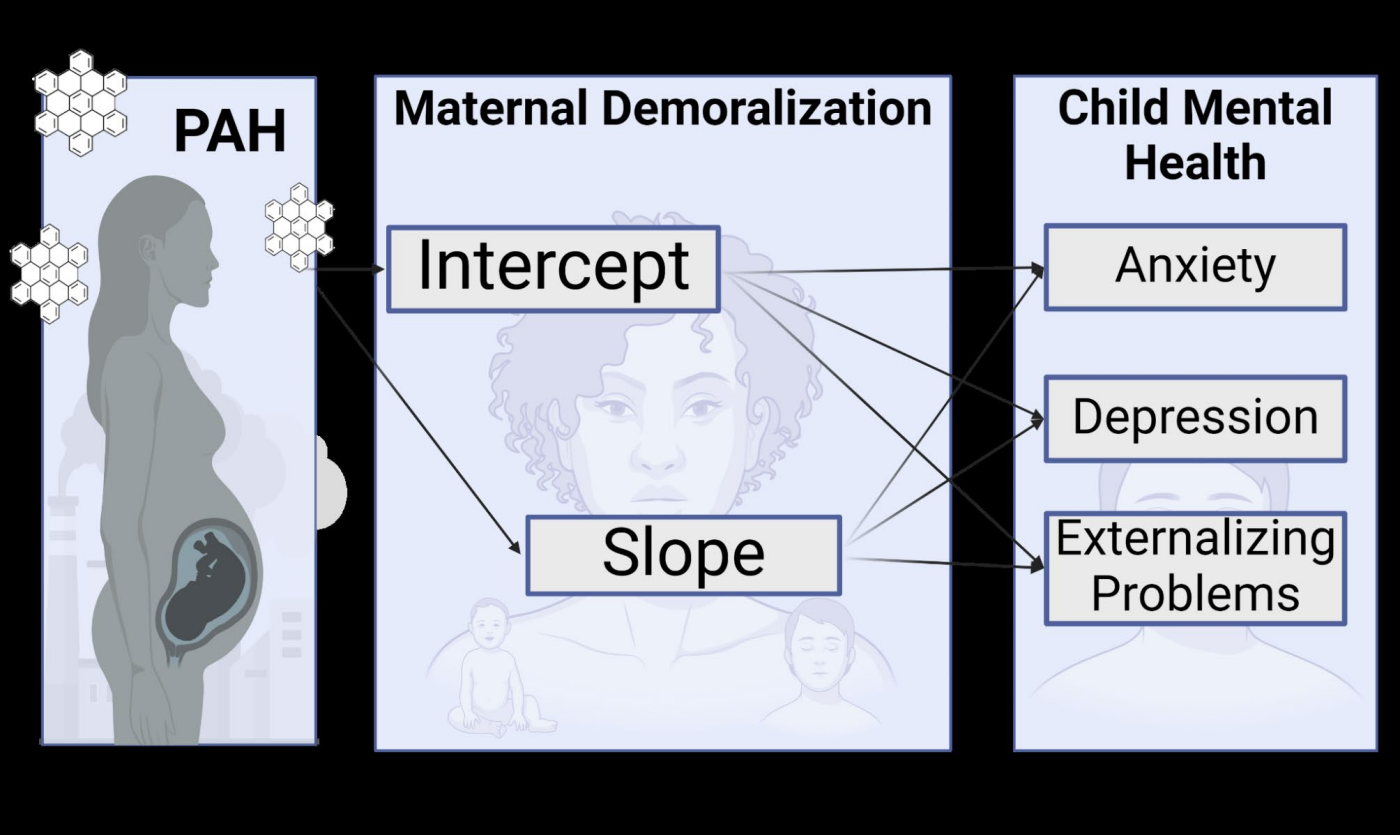
Conceptual model

Next, adolescent’s self-reported mental health symptoms at age 14 were added to the structural equation model described above. We evaluated three models, one for each symptom group (anxiety, depression, externalizing problems; Fig. 1). Covariates known to be associated with adolescent mental health outcomes were included in the second step of the model, specifically: maternal years of education as a proxy for socioeconomic status and baby’s sex at birth [96, 97] (See supplementary methods for details). To determine if effects persisted over time, confirmatory analyses used adolescent self-report data from age 16 (RCMAS and YSR) in each of the above models.

## Results

### Participants

Tables 1 and 2 presents demographic information for the study sample. Mothers included in this study did not differ from those who did not have available data on age or maternal education. Black mothers were more likely than Latiné mothers to be excluded from the study (χ^2^ = 5.24, *p* = 0.02). Adolescents with age 14 self-report measures did not differ than those without self-report measures on maternal PAH-DNA adducts, maternal nativity status, the presence of a smoker in the home, birth season (i.e., heat season), ethnoracial group, gestational age, maternal age at birth, maternal years of education, or maternal IQ.

**Table 2:** Age 14 mental health self-report measures

Scale	*N*	Mean (SD)	Min	Max
RCMAS	354	8.86 (5.99)	0	28
CESD	355	16.88 (6.49)	0	45
YSR externalizing problems	303	49.97 (9.08)	29	76

RCMAS revised children’s manifest anxiety scale, CESD center for epidemiologic studies depression scale, YSR youth self-report

Adolescents with age 14 self-report measures were more likely to be biologically female (χ^2^ = 6.28, *p* = 0.01). Adolescents with age 14 self-report measures did not differ than those without self-report measures maternal nativity, the presence of a smoker in the home, birth season (i.e., heat season), sex at birth, ethnoracial group, gestational age, maternal years of education, or maternal IQ. Mothers of adolescents with age 16 self-report measures were more likely to be younger (t = -2.24, *p* = 0.03) and less likely to be exposed to PAH than adolescents without age 16 self-report data.

### PAH exposure is associated with higher prenatal maternal demoralization

The latent growth model indicated good model fit (RMSEA = 0.06, RMSEA p-value < 0.01, CFI = 0.9, SRMR = 0.05; details in Supplementary Results). Presence (v. absence) of PAH-DNA adducts was associated with higher prenatal maternal demoralization (intercept β = 3.02, SE = 1.51, *p* = 0.04; Table 3) but not the trajectory of change in maternal demoralization over time (slope β = -0.01, SE = 0.01, *p* = NS; Table 3). Main effects of race (β = -4.19, SE = 2.12, *p* = 0.04; Table 5) and a smoker living at home (β = 3.54, SE = 0.78, *p* < 0.01; Table 5) were also significant. Latiné mothers reported higher prenatal demoralization, as did mothers living with a smoker, who also reported smaller decreases in demoralization over time (β = -0.01, SE = 0.01, *p* = 0.03, Table 5). Across the entire sample, the average predicted demoralization score was approximately 39 (β = 38.66; *p*_*intercept*_<0.01), representing moderate levels of demoralization (total score range = 0-108), which declined over time (β=-0.08; *p*_*slope*_=0.03). Finally, the covariance of the intercept and the slope differed from zero (β=-0.33; *p* < 0.01), indicating that prenatal demoralization scores correlated with the change in demoralization over time.

**Table 3:** Latent growth curve model results

Outcome:	Demoralization intercept			Demoralization slope		
Predictor	Coefficient	z-value	p-value	Coefficient	z-value	p-value
Maternal PAH-DNA adducts	3.03	2.01	0.04	-0.01	-0.80	0.42
Gestational age	-1.09	-1.43	0.15	0.004	0.74	0.46
Maternal age at birth	1.35	1.62	0.11	0.01	1.02	0.31
Maternal nativity	-3.61	-1.62	0.11	0.02	1.51	0.13
Maternal prenatal years of education	-0.70	-0.81	0.42	-0.01	-0.85	0.39
Smoker at home	3.54	4.51	0.00	-0.01	-2.16	0.03
Heat season	0.25	0.16	0.87	-0.002	-0.20	0.84
Maternal intelligence	-0.44	-0.51	0.61	0.003	0.55	0.58
Maternal ethnoracial identification	-4.23	-2.00	0.04	0.02	1.37	0.17

PAH polycyclic aromatic hydrocarbons

### Maternal demoralization is associated with higher adolescent self-reported mental health symptoms

To test the association between maternal demoralization (prenatal and change over time) and adolescent mental health symptoms, adolescent self-report symptoms were added to the structural equation model described above. The latent growth curve describing maternal demoralization did not meaningfully change with the addition of adolescent mental health symptoms (see Supplement for details).

### Maternal demoralization is associated with greater adolescent self-reported anxiety

Main effects of prenatal maternal demoralization (β = 0.01, SE = 0.004, *p* < 0.01) and the trajectory of maternal demoralization over time (β = 2.27, SE = 0.93, *p* = 0.02) were associated with greater adolescent self-reported anxiety symptoms. Girls reported more anxiety than boys (β = -0.29, SE = 0.07, *p* < 0.01). No other significant findings were observed (Table 4). Results were similar at age 16 (Supplemental Table S1). Supplementary analysis determined that there was no direct effect of PAH-DNA adducts on child reported anxiety at age 14 (Supplemental Table S4).

**Table 4:** Latent growth curve model results - adolescent anxiety at Age 14

Predictor	Coefficient	z-value	*p*-value
Demoralization intercept	0.01	3.78	0.00
Demoralization slope	2.27	2.44	0.02
Maternal prenatal years of education	0.03	0.46	0.65
Child sex at birth	-0.29	-4.01	0.00

### Maternal demoralization is associated with greater adolescent self-reported depression

Main effects of prenatal maternal demoralization were associated with greater adolescent self-reported depression symptoms (β = 0.01, SE = 0.004, *p* < 0.01). Girls reported more depression than boys (β = -0.27, SE = 0.08, *p* < 0.01). No other significant findings were observed (Table 5). Supplementary analysis determined that there was no direct effect of PAH-DNA adducts on child reported depression at age 14 (Supplemental Table S5).

**Table 5:** Latent growth curve model results - adolescent depression at Age 14

Predictor	Coefficient	z-value	*p*-value
Demoralization intercept	0.01	3.39	0.001
Demoralization slope	1.26	1.29	0.20
Maternal prenatal years of education	-0.04	-0.59	0.56
Child sex at birth	-0.27	-3.48	0.001

PAH polycyclic aromatic hydrocarbons

### Maternal demoralization is associated with greater adolescent self-reported externalizing problems

Main effects of prenatal maternal demoralization (β = 0.01, SE = 0.004, *p* = 0.03) and the trajectory of maternal demoralization over time were associated with greater adolescent self-reported externalizing symptoms (β = 3.59, SE = 1.06, *p* < 0.01). Girls reported higher externalizing scores than boys (β = -0.16, SE = -2.10, *p* = 0.04). No other significant findings were observed (Table 6). Results were similar at age 16 (Supplemental Table S2). Supplementary analysis determined that there was no direct effect of PAH-DNA adducts on child reported externalizing problems at age 14 (Supplemental Table S6).

**Table 6:** Latent growth curve model results - adolescent externalizing problems at Age 14

Predictor	Coefficient	z-value	*p*-value
Demoralization intercept	0.01	2.18	0.03
Demoralization slope	3.5	3.49	0.00
Maternal prenatal years of education	0.09	1.34	0.18
Child sex at birth	-0.16	-2.10	0.04

## Discussion

The current study examined associations between prenatal PAH exposure, trajectories of maternal demoralization, and adolescent self-reported anxiety, depression, and externalizing problems. Higher prenatal PAH exposure was associated with higher prenatal maternal demoralization, which predicted higher adolescent self-reported anxiety, depression, and externalizing problems. Additionally, maternal demoralization declined over time, with slower declines predicting higher adolescent self-reported anxiety and externalizing problems. Within an intergenerational transmission of mental health framework, our findings suggest that PAH exposure during pregnancy affects concurrent and long term maternal mental health, which in turn influences mental health outcomes for youth well into adolescence.

### Prenatal PAH exposure associates with maternal demoralization during pregnancy

PAH exposure during pregnancy was associated with higher self-reported prenatal maternal demoralization. Our work extends prior findings showing associations of concurrent PAH exposure with depression symptoms and psychological distress, and suggests that PAH exposure may also increase risk for prepartum demoralization. Translational models offer several mechanisms through which PAHs may affect adult mental and behavioral health, including direct assault and damage on neurological tissues [98, 99], oxidative stress associated with PAH metabolism [99], and the inhibition of essential enzymes to result in downstream, cascading effects on cognition and behavior particularly in systems reliant on dopamine [100–102], a neurotransmitter closely linked to many mental health problems [103, 104]. Critically, biological changes such as changes in blood volume and immune system function associated with pregnancy may increase vulnerability to the effects of PAH exposure through inflammatory processes (for review: [104]). As such, it is possible that PAH exposure during pregnancy (versus not during pregnancy) differentially affects biological pathways in ways that magnify psychological distress and demoralization. Alternatively, pregnant people who are experiencing higher demoralization may also experience higher PAH exposure as a function of structural factors in the built environment.

Prenatal PAH exposure did not moderate trajectories of maternal demoralization. In 2010, public policy changes were enacted to improve air quality in New York City, resulting in decreases in PAH exposure for individuals in our cohort catchment area [105]. Thus, it is possible that improvements in outdoor air quality (i.e., less concurrent PAH exposure) were associated with improvements in concurrent maternal demoralization. However, we only measured PAH exposure in mothers during the pregnancy period, and so we cannot directly test this hypothesis. Future studies could measure PAH exposure across the perinatal period to determine if changes in exposure are associated with changes in demoralization over time. If reductions in PAH exposure due to public policy change resulted in reduced maternal psychological distress, this supports the urgent need for further policy change directed at reducing PAH exposure for vulnerable populations.

### Maternal demoralization predicts adolescent self-reported mental and behavioral health

Maternal demoralization during pregnancy and over the course of childhood predicted higher adolescent self-reported mental health symptoms. Notably, higher PAH-related persistent demoralization was associated with youth self-reported anxiety but not depression symptoms. Our work aligns with and extends prior findings showing that maternal psychological distress during pregnancy is associated with youth internalizing symptoms and may be attributable in part to PAH exposure as well as to maternal depression [106, 107]. Consistent with robustly reported sex differences in internalizing symptoms, females in our sample reported higher anxiety and depression symptoms than males [108, 109]; across males and females the average reported depression score was in the at risk range [109] pointing to the vulnerability of youth living in these contexts. In sum, elevated PAH exposure prenatally may set mothers on a path towards persistently poor mental health, which in turn predicts greater self-reported anxiety and depression symptoms in their children.

Maternal demoralization during pregnancy and over time also predicted higher self-reported externalizing problems in adolescents. Prior findings in this cohort and others indicate associations between prenatal PAH exposure and maternal ratings of externalizing behavior problems at age 9, including inattention and hyperactivity [49, 110]. Such findings could indicate report bias such that PAH-related effects on maternal mental health influence maternal ratings of children’s behavior. Our findings that maternal demoralization is associated with youth’s own perception of their ability to regulate their emotions and behaviors suggests that reported bias does not explain prior findings and instead points to a complex interplay between maternal chemical exposure, maternal mental health, and youth development.

### Summary and limitations

Children from areas with higher poverty are more likely to face higher rates of exposure to both chemical neurotoxicants and social stressors [17], including but not limited to maternal demoralization. The specific mechanisms responsible for the interaction of maternal demoralization and PAH are yet to be elucidated. Potential physiological factors range from maternal cortisol transfer to the fetus [111–113], mRNA expression [112] and inflammatory pathway dysregulation [114]. Social factors include differences in home environment [115], parenting skills [116–118], and lack of material resources [97–99]. Future studies are necessary to better understand the biological and social underpinnings of the intergenerational transmission of PAH-related emotional and behavioral dysregulation. Our findings are consistent with and extend the existing literature indicating that prenatal exposure to air pollution predisposes both elevated, persistent maternal distress and mental health problems in adolescents.

Our findings should be considered in light of a few limitations. First, maternal demoralization is a fairly understudied construct separate from psychological or psychiatric symptoms. Because our study does not contain a clinical measure of maternal anxiety or depression symptoms we were unable to explore this further but future work can delineate the boundaries and characterization of maternal demoralization. Next, because our cohort is a community sample and is not weighted for clinical outcomes, our findings are not generalizable to a population of youth with clinically significant mental health outcomes. Future studies should examine these trajectories in a cohort weighted for clinical symptoms in order to assess these effects. As described above, we did not measure concurrent PAH exposure in mothers or children across child development which would allow further understanding of critical timepoints for effective intervention and exposure reduction. Finally, data missingness may limit generalizability.

## Conclusions

PAH exposure has detrimental impacts on both maternal and child mental health. Our study extends previous work by examining trajectories of PAH-related maternal demoralization symptoms and adolescent mental health. Our findings support an intergenerational transmission of PAH-related psychological distress such that mothers exposed to PAH during pregnancy are more likely to demonstrate higher demoralization over time and in turn their children report higher mental and behavioral health symptoms. These findings support public policy changes aimed at reducing PAH exposure during pregnancy as well as the need for longer term screening and intervention for maternal psychological distress. Early identification and intervention for maternal psychological distress may therefore reduce the burden of psychological distress in adolescents.

## Supplementary Information

Supplementary material 1

## Data Availability

This study leveraged data from the CCCEH Mothers and Newborns prospective, longitudinal birth cohort. Data is available upon written request.

## Abbreviations

PAH: Polycyclic aromatic hydrocarbons
DNA: Deoxyribonucleic acid
CCCEH: Columbia center for children’s environmental health
PERI-D: Psychiatric epidemiology research instrument–demoralization
RCMAS: Revised children’s manifest anxiety scale
CES-D: Center for epidemiologic studies depression scale
YSR: Youth self-report of the children’s behavior checklist
CFI: Comparative fit index
RMSEA: Root mean square error of approximation
SRMR: Standardized root mean square residual
TONI: Test of nonverbal intelligence

## References

[1] Trentacosta CJ, Mulligan DJ. New directions in understanding the role of environmental contaminants in child development: four themes. New Dir Child Adolesc Dev. 2020;2020(172):39–51. 32920950 10.1002/cad.20363 PMC8189654

[2] Boström C-E, Gerde P, Hanberg A, Jernström B, Johansson C, Kyrklund T, et al. Cancer risk assessment, indicators, and guidelines for polycyclic aromatic hydrocarbons in the ambient air. Environ Health Perspect. 2002;110(3suppl 3):451–88. 12060843 10.1289/ehp.110-1241197 PMC1241197

[3] Choi H, Harrison R, Komulainen H, Delgado Saborit JM. Polycyclic aromatic hydrocarbons. World Health Organization; 2010.

[4] Baek SO, Field RA, Goldstone ME, Kirk PW, Lester JN, Perry R. A review of atmospheric polycyclic aromatic hydrocarbons: sources, fate and behavior. Water Air Soil Pollut. 1991;60(3):279–300. 10.1007/BF00282628

[5] Zhu L, Takahashi Y, Amagai T, Matsushita H. Highly sensitive automatic analysis of polycyclic aromatic hydrocarbons in indoor and outdoor air. Talanta. 1997;45(1):113–8. 18966986 10.1016/S0039-9140(97)00109-4

[6] Fromme H, Oddoy A, Piloty M, Krause M, Lahrz T. Polycyclic aromatic hydrocarbons (PAH) and diesel engine emission (elemental carbon) inside a car and a subway train. Sci Total Environ. 1998;217(1–2):165–73. 9695180 10.1016/S0048-9697(98)00189-2

[7] Li C-S, Ro Y-S. Indoor characteristics of polycyclic aromatic hydrocarbons in the urban atmosphere of Taipei. Atmos Environ (1994). 2000;34(4):611–20. 10.1016/S1352-2310(99)00171-5

[8] Lung S-CC, Kao M-C, Hu S-C. Contribution of incense burning to indoor PM10 and particle-bound polycyclic aromatic hydrocarbons under two ventilation conditions. Indoor Air. 2003;13(2):194–9. 12756013 10.1034/j.1600-0668.2003.00197.x

[9] Lau C, Fiedler H, Hutzinger O, Schwind KH, Hosseinpour J. Levels of selected organic compounds in materials for candle production and human exposure to candle emissions. Chemosphere. 1997;34(5–7):1623–30. 9134692 10.1016/S0045-6535(97)00458-X

[10] U.S. Environmental Protection Agency (EPA). Exposure factors handbook. 2011th ed. Washington, DC: National Center for Environmental Assessment; 2011.

[11] Agency for Toxic Substances and Disease Registry. Toxicological profile for polycyclic aromatic hydrocarbons (PAHs) (update). Atlanta, GA: US Department of Health and Human Services. 38091452

[12] Chen Z, Salam MT, Eckel SP, Breton CV, Gilliland FD. Chronic effects of air pollution on respiratory health in Southern California children: findings from the Southern California Children’s Health Study. J Thorac Dis. 2015;7(1):46–58. 25694817 10.3978/j.issn.2072-1439.2014.12.20 PMC4311073

[13] Air pollution and child health: prescribing clean air [Internet]. World Health Organization. 2018 [cited 2024 Aug 16]. https://www.who.int/publications/i/item/WHO-CED-PHE-18-01

[14] Ghosh R, Causey K, Burkart K, Wozniak S, Cohen A, Brauer M. Ambient and household PM2.5 pollution and adverse perinatal outcomes: a meta-regression and analysis of attributable global burden for 204 countries and territories. PLoS Med. 2021;18(9):e1003718. 34582444 10.1371/journal.pmed.1003718 PMC8478226

[15] Mendoza-Sanchez I, Uwak I, Myatt L, Van Cleve A, Pulczinski JC, Rychlik KA, et al. Maternal exposure to polycyclic aromatic hydrocarbons in South Texas, evaluation of silicone wristbands as personal passive samplers. J Expo Sci Environ Epidemiol. 2022;32(2):280–8. 34131287 10.1038/s41370-021-00348-y PMC8920889

[16] Dai Y, Xu X, Huo X, Faas MM. Effects of polycyclic aromatic hydrocarbons (PAHs) on pregnancy, placenta, and placental trophoblasts. Ecotoxicol Environ Saf. 2023;262(115314):115314. 37536008 10.1016/j.ecoenv.2023.115314

[17] Hajat A, Hsia C, O’Neill MS. Socioeconomic disparities and air pollution exposure: a global review. Curr Environ Health Rep. 2015;2(4):440–50. 26381684 10.1007/s40572-015-0069-5 PMC4626327

[18] Choi H, Harrison R, Komulainen H, Delgado Saborit JM. In: Leger A, D’Hendecourt L, Boccara N, editors. Polycyclic aromatic hydrocarbons and astrophysics. Dordrecht, Netherlands: Springer; 2011. p. 402.

[19] Rahman HH, Niemann D, Munson-McGee SH. Association among urinary polycyclic aromatic hydrocarbons and depression: a cross-sectional study from NHANES 2015–2016. Environ Sci Pollut Res Int. 2022;29(9):13089–97. 34569004 10.1007/s11356-021-16692-3

[20] Zhang L, Sun J, Zhang D. The relationship between urine polycyclic aromatic hydrocarbons and depressive symptoms in American adults. J Affect Disord. 2021;292:227–33. 34130188 10.1016/j.jad.2021.05.097

[21] Zhen H, Zhang F, Cheng H, Hu F, Jia Y, Hou Y, et al. Association of polycyclic aromatic hydrocarbons exposure with child neurodevelopment and adult emotional disorders: a meta-analysis study. Ecotoxicol Environ Saf. 2023;255:114770. 36931089 10.1016/j.ecoenv.2023.114770

[22] Rojas GA, Saavedra N, Saavedra K, Hevia M, Morales C, Lanas F, et al. Polycyclic aromatic hydrocarbons (PAHs) exposure triggers inflammation and endothelial dysfunction in BALB/c mice: a pilot study. Toxics. 2022;10(9):497. 36136462 10.3390/toxics10090497 PMC9504903

[23] Bauer AK, Velmurugan K, Plöttner S, Siegrist KJ, Romo D, Welge P, et al. Environmentally prevalent polycyclic aromatic hydrocarbons can elicit co-carcinogenic properties in an in vitro murine lung epithelial cell model. Arch Toxicol. 2018;92(3):1311–22. 29170806 10.1007/s00204-017-2124-5 PMC5866845

[24] Bright A, Li F, Movahed M, Shi H, Xue B. Chronic exposure to low-molecular-weight polycyclic aromatic hydrocarbons promotes lipid accumulation and metabolic inflammation. Biomolecules. 2023;13(2):196. 36830566 10.3390/biom13020196 PMC9953192

[25] Varshavsky J, Smith A, Wang A, Hom E, Izano M, Huang H, et al. Heightened susceptibility: a review of how pregnancy and chemical exposures influence maternal health. Reprod Toxicol. 2020;92:14–56. 31055053 10.1016/j.reprotox.2019.04.004 PMC6824944

[26] Perera FP, Tang D, Wang S, Vishnevetsky J, Zhang B, Diaz D, et al. Prenatal polycyclic aromatic hydrocarbon (PAH) exposure and child behavior at age 6–7 years. Environ Health Perspect. 2012;120(6):921–6. 22440811 10.1289/ehp.1104315 PMC3385432

[27] Allen JL, Liu X, Pelkowski S, Palmer B, Conrad K, Oberdörster G, et al. Early postnatal exposure to ultrafine particulate matter air pollution: persistent ventriculomegaly, neurochemical disruption, and glial activation preferentially in male mice. Environ Health Perspect. 2014;122(9):939–45. 24901756 10.1289/ehp.1307984 PMC4154219

[28] Klocke C, Allen JL, Sobolewski M, Mayer-Pröschel M, Blum JL, Lauterstein D, et al. Neuropathological consequences of gestational exposure to concentrated ambient fine and ultrafine particles in the mouse. Toxicol Sci. 2017;156(2):492–508. 28087836 10.1093/toxsci/kfx010 PMC6074840

[29] Margolis AE, Cohen JW, Ramphal B, Thomas L, Rauh V, Herbstman J, et al. Prenatal exposure to air pollution and early-life stress effects on hippocampal subregional volumes and associations with visuospatial reasoning. Biol Psychiatry Glob Open Sci. 2022;2(3):292–300. 35978944 10.1016/j.bpsgos.2022.05.003 PMC9380862

[30] Tachibana K, Takayanagi K, Akimoto A, Ueda K, Shinkai Y, Umezawa M, et al. Prenatal diesel exhaust exposure disrupts the DNA methylation profile in the brain of mouse offspring. J Toxicol Sci. 2015;40(1):1–11. 25560391 10.2131/jts.40.1

[31] Bolton JL, Huff NC, Smith SH, Mason SN, Foster WM, Auten RL, et al. Maternal stress and effects of prenatal air pollution on offspring mental health outcomes in mice. Environ Health Perspect. 2013;121(9):1075–82. 23823752 10.1289/ehp.1306560 PMC3764088

[32] Goodman SH, Rouse MH, Connell AM, Broth MR, Hall CM, Heyward D. Maternal depression and child psychopathology: a meta-analytic review. Clin Child Fam Psychol Rev. 2011;14(1):1–27. 21052833 10.1007/s10567-010-0080-1

[33] Szekely E, Neumann A, Sallis H, Jolicoeur-Martineau A, Verhulst FC, Meaney MJ, et al. Maternal prenatal mood, pregnancy-specific worries, and early child psychopathology: findings from the DREAM BIG consortium. J Am Acad Child Adolesc Psychiatry. 2021;60(1):186–97. 32278003 10.1016/j.jaac.2020.02.017

[34] Zhang H, Lee ZX, White T, Qiu A. Parental and social factors in relation to child psychopathology, behavior, and cognitive function. Transl Psychiatry. 2020;10(1):80. 32102994 10.1038/s41398-020-0761-6 PMC7044210

[35] Goodman SH, Simon HFM, Shamblaw AL, Kim CY. Parenting as a mediator of associations between depression in mothers and children’s functioning: a systematic review and meta-analysis. Clin Child Fam Psychol Rev. 2020;23(4):427–60. 32734498 10.1007/s10567-020-00322-4

[36] Barnes J, Theule J. Maternal depression and infant attachment security: a meta-analysis. Infant Ment Health J. 2019;40(6):817–34. 31415711 10.1002/imhj.21812

[37] Ahun MN, Côté SM. Maternal depressive symptoms and early childhood cognitive development: a review of putative environmental mediators. Arch Womens Ment Health. 2019;22(1):15–24. 29876681 10.1007/s00737-018-0870-x

[38] Sawyer KM, Zunszain PA, Dazzan P, Pariante CM. Intergenerational transmission of depression: clinical observations and molecular mechanisms. Mol Psychiatry. 2019;24(8):1157–77. 30283036 10.1038/s41380-018-0265-4

[39] Paananen R, Tuulio-Henriksson A, Merikukka M, Gissler M. Intergenerational transmission of psychiatric disorders: the 1987 Finnish birth cohort study. Eur Child Adolesc Psychiatry. 2021;30(3):381–9. 32270343 10.1007/s00787-020-01524-5

[40] Duarte CS, Monk C, Weissman MM, Posner J. Intergenerational psychiatry: a new look at a powerful perspective. World Psychiatry. 2020;19(2):175–6. 32394546 10.1002/wps.20733 PMC7214952

[41] Moog NK, Cummings PD, Jackson KL, Aschner JL, Barrett ES, Bastain TM, et al. Intergenerational transmission of the effects of maternal exposure to childhood maltreatment in the USA: a retrospective cohort study. Lancet Public Health. 2023;8(3):e226–37. 36841563 10.1016/S2468-2667(23)00025-7 PMC9982823

[42] Barat S, Ghanbarpour A, Mirtabar SM, Kheirkhah F, Basirat Z, Shirafkan H et al. Psychological distress in pregnancy and postpartum: a cross-sectional study of Babol pregnancy mental health registry. BMC Pregnancy Childbirth [Internet]. 2023 Nov 14 [cited 2024 Aug 16];23(1). https://www.ncbi.nlm.nih.gov/pmc/articles/PMC10648632/10.1186/s12884-023-06024-3 PMC10648632 37964209

[43] de Figueiredo JM. Depression and demoralization: phenomenologic differences and research perspectives. Compr Psychiatry. 1993;34(5):308–11. 8306640 10.1016/0010-440X(93)90016-W

[44] Frank JD. Psychotherapy: the restoration of morale. Am J Psychiatry. 1974;131(3):271–4. 4812687 10.1176/ajp.131.3.271

[45] Liu ST, Wu X, Wang N, Zhao QQ, Xiao L, Fang CK, et al. Serial multiple mediation of demoralization and depression in the relationship between hopelessness and suicidal ideation. Psychooncology. 2020;29(8):1321–8. 32539164 10.1002/pon.5439

[46] Costanza A, Baertschi M, Richard-Lepouriel H, Weber K, Berardelli I, Pompili M, et al. Demoralization and its relationship with depression and hopelessness in suicidal patients attending an emergency department. Int J Environ Res Public Health. 2020. 10.3390/ijerph17072232. 32225017 10.3390/ijerph17072232 PMC7177663

[47] Tecuta L, Tomba E, Grandi S, Fava GA. Demoralization: a systematic review on its clinical characterization. Psychol Med. 2015;45(4):673–91. 25032712 10.1017/S0033291714001597

[48] Reyes M, Perzanowski MS, Whyatt RM, Kelvin EA, Rundle AG, Diaz DM, et al. Relationship between maternal demoralization, wheeze, and immunoglobulin E among inner-city children. Ann Allergy Asthma Immunol. 2011;107(1):42–e491. 21704884 10.1016/j.anai.2011.03.004 PMC3135280

[49] Perera FP, Wang S, Rauh V, Zhou H, Stigter L, Camann D, et al. Prenatal exposure to air pollution, maternal psychological distress, and child behavior. Pediatrics. 2013;132(5):e1284–94. 24101766 10.1542/peds.2012-3844 PMC3813389

[50] DeSerisy M, Cohen JW, Dworkin JD, Stingone JA, Ramphal B, Herbstman JB, et al. Early life stress, prenatal secondhand smoke exposure, and the development of internalizing symptoms across childhood. Environ Health. 2023;22(1):58. 37620883 10.1186/s12940-023-01012-8 PMC10463722

[51] Greenwood PB, DeSerisy M, Koe E, Rodriguez E, Salas L, Perera FP, et al. Combined and sequential exposure to prenatal second hand smoke and postnatal maternal distress is associated with cingulo-opercular global efficiency and attention problems in school-age children. Neurotoxicol Teratol. 2024;102(107338):107338. 38431065 10.1016/j.ntt.2024.107338 PMC11781759

[52] Hipke KN, Wolchik SA, Sandler IN, Braver SL. Predictors of children’s intervention-Induced Resilience in a parenting program for divorced mothers. Fam Relat. 2002;51(2):121–9. 10.1111/j.1741-3729.2002.00121.x

[53] Kessler RC, McRae JA Jr. Trends in the relationship between sex and psychological distress: 1957–1976. Am Sociol Rev. 1981;46(4):443–52. 11630842 10.2307/2095263

[54] Radloff L. Sex differences in depression. Sex Roles [Internet]. 1975;1(3). https://idp.springer.com/authorize/casa?redirect_uri=https://link.springer.com/article/10.1007/BF00287373&casa_token=w8-cK8ew2XQAAAAA:7pPJK-p_p5NPRhqwDNCMIBdHv7T6KWZ1Srl4yn9R8pPUCUU_wzwgcoLa3J8BX4-3NuijBZEg3CGjocOw

[55] Hess RD, Camara KA. Post-divorce family relationships as mediating factors in the consequences of divorce for children. J Soc Issues. 1979;35(4):79–96. 10.1111/j.1540-4560.1979.tb00814.x

[56] Cohen P, Johnson J, Lewis SA, Brook JS. Single parenthood and employment double Jeopardy? In: Eckenrode J, Gore S, editors. Stress between work and family. Boston, MA: Springer US; 1990. pp. 117–32.

[57] Davis DA, Bortolato M, Godar SC, Sander TK, Iwata N, Pakbin P, et al. Prenatal exposure to urban air nanoparticles in mice causes altered neuronal differentiation and depression-like responses. PLoS ONE. 2013;8(5):e64128. 23734187 10.1371/journal.pone.0064128 PMC3667185

[58] Fonken LK, Xu X, Weil ZM, Chen G, Sun Q, Rajagopalan S, et al. Air pollution impairs cognition, provokes depressive-like behaviors and alters hippocampal cytokine expression and morphology. Mol Psychiatry. 2011;16(10):987–95. 21727897 10.1038/mp.2011.76 PMC3270364

[59] Win-Shwe T-T, Fujitani Y, Kyi-Tha-Thu C, Furuyama A, Michikawa T, Tsukahara S, et al. Effects of diesel engine exhaust origin secondary organic aerosols on novel object recognition ability and maternal behavior in BALB/c mice. Int J Environ Res Public Health. 2014;11(11):11286–307. 25361045 10.3390/ijerph111111286 PMC4245613

[60] Perera FP, Rauh V, Whyatt RM, Tsai W-Y, Tang D, Diaz D, et al. Effect of prenatal exposure to airborne polycyclic aromatic hydrocarbons on neurodevelopment in the first 3 years of life among inner-city children. Environ Health Perspect. 2006;114(8):1287–92. 16882541 10.1289/ehp.9084 PMC1551985

[61] Dohrenwend BP, Shrout PE, Egri G, Mendelsohn FS. Nonspecific psychological distress and other dimensions of psychopathology. Measures for use in the general population. Arch Gen Psychiatry. 1980;37(11):1229–36. 7436685 10.1001/archpsyc.1980.01780240027003

[62] Buckingham-Howes S, Oberlander SE, Wang Y, Black MM. Early maternal depressive symptom trajectories: associations with 7-year maternal depressive symptoms and child behavior. J Fam Psychol. 2017;31(4):387–97. 27632350 10.1037/fam0000242

[63] Cents RAM, Diamantopoulou S, Hudziak JJ, Jaddoe VWV, Hofman A, Verhulst FC, et al. Trajectories of maternal depressive symptoms predict child problem behaviour: the Generation R study. Psychol Med. 2013;43(1):13–25. 22490169 10.1017/S0033291712000657

[64] van der Waerden J, Galéra C, Larroque B, Saurel-Cubizolles M-J, Sutter-Dallay A-L, Melchior M, et al. Maternal Depression trajectories and children’s Behavior at Age 5 years. J Pediatr. 2015;166(6):1440–e81. 25866387 10.1016/j.jpeds.2015.03.002

[65] Putnick DL, Sundaram R, Bell EM, Ghassabian A, Goldstein RB, Robinson SL, et al. Trajectories of maternal postpartum depressive symptoms. Pediatrics. 2020. 10.1542/peds.2020-0857. 33109744 10.1542/peds.2020-0857 PMC7772818

[66] Tearne JE, Allen KL, Herbison CE, Lawrence D, Whitehouse AJO, Sawyer MG, et al. The association between prenatal environment and children’s mental health trajectories from 2 to 14 years. Eur Child Adolesc Psychiatry. 2015;24(9):1015–24. 25431038 10.1007/s00787-014-0651-7

[67] De Los Reyes A, Goodman KL, Kliewer W, Reid-Quiñones K. Whose depression relates to discrepancies? Testing relations between informant characteristics and informant discrepancies from both informants’ perspectives. Psychol Assess. 2008;20(2):139–49. 18557691 10.1037/1040-3590.20.2.139 PMC2610408

[68] Chilcoat HD, Breslau N. Does psychiatric history bias mothers’ reports? An application of a new analytic approach. J Am Acad Child Adolesc Psychiatry. 1997;36(7):971–9. 9204676 10.1097/00004583-199707000-00020

[69] Gartstein MA, Bridgett DJ, Dishion TJ, Kaufman NK. Depressed mood and maternal report of child behavior problems: another look at the depression-distortion hypothesis. J Appl Dev Psychol. 2009;30(2):149–60. 20161323 10.1016/j.appdev.2008.12.001 PMC2678740

[70] Müller JM, Achtergarde S, Furniss T. The influence of maternal psychopathology on ratings of child psychiatric symptoms: an SEM analysis on cross-informant agreement. Eur Child Adolesc Psychiatry. 2011;20(5):241–52. 21416135 10.1007/s00787-011-0168-2

[71] Cantwell DP, Lewinsohn PM, Rohde P, Seeley JR. Correspondence between adolescent report and parent report of psychiatric diagnostic data. J Am Acad Child Adolesc Psychiatry. 1997;36(5):610–9. 9136495 10.1097/00004583-199705000-00011

[72] Reynolds CR, Richmond BO. Revised children’s manifest anxiety Scale Manual. Los Angeles, CA: Western Psychological; 1985.

[73] White KS, Farrell AD. Structure of anxiety symptoms in urban children: competing factor models of the revised children’s manifest anxiety scale. J Consult Clin Psychol. 2001;69(2):333–7. 11393610 10.1037/0022-006X.69.2.333

[74] Reynolds CR, Richmond BO. What I think and feel: a revised measure of children’s manifest anxiety. J Abnorm Child Psychol. 1978;6(2):271–80. 670592 10.1007/BF00919131

[75] Stallard P, Velleman R, Langsford J, Baldwin S. Coping and psychological distress in children involved in road traffic accidents. Br J Clin Psychol. 2001;40(2):197–208. 11446241 10.1348/014466501163643

[76] Radloff LS. The CES-D scale: a self report depression scale for research in the general population. Appl Psychol Measurements. 1977;1:385–401. 10.1177/014662167700100306

[77] Weissman MM, Orvaschel H, Padian N. Children’s symptom and social functioning self-report scales. Comparison of mothers’ and children’s reports. J Nerv Ment Dis. 1980;168(12):736–40. 7452212 10.1097/00005053-198012000-00005

[78] Achenbach TM, Rescorla L. Manual for the ASEBA (Achenbach System of empirically-based Assessment) school-age forms and profiles. Burlington: Research Center for Children, Youth, and Families, Department of Psychiatry, University of Vermont; 2001.

[79] Mooney LA, Santella RM, Covey L, Jeffrey AM, Bigbee W, Randall MC, et al. Decline of DNA damage and other biomarkers in peripheral blood following smoking cessation. Cancer Epidemiol Biomarkers Prev. 1995;4(6):627–34. 8547829

[80] Stéphan-Blanchard E, Chardon K, Telliez F, Arnould J-P, Léké A, Ammari M, et al. Are Benzo[a]pyrene–DNA adducts an accurate biomarker of long-term in utero exposure to smoking? Ther Drug Monit. 2011;33(3):329–35. 21544016 10.1097/FTD.0b013e31821bb660

[81] Cheng T, Lam AK, Gopalan V. Diet derived polycyclic aromatic hydrocarbons and its pathogenic roles in colorectal carcinogenesis. Crit Rev Oncol Hematol. 2021;168(103522):103522. 34748942 10.1016/j.critrevonc.2021.103522

[82] Alvarado-Cruz I, Alegría-Torres JA, Montes-Castro N, Jiménez-Garza O, Quintanilla-Vega B. Environmental epigenetic changes, as risk factors for the development of diseases in children: a systematic review. Ann Glob Health. 2018;84(2):212–24. 30873799 10.29024/aogh.909 PMC6748183

[83] Bukowska B, Sicińska P. Influence of Benzo(a)pyrene on different epigenetic processes. Int J Mol Sci. 2021;22(24):13453. 34948252 10.3390/ijms222413453 PMC8707600

[84] Herbstman JB, Tang D, Zhu D, Qu L, Sjödin A, Li Z, et al. Prenatal exposure to polycyclic aromatic hydrocarbons, benzo[a]pyrene-DNA adducts, and genomic DNA methylation in cord blood. Environ Health Perspect. 2012;120(5):733–8. 22256332 10.1289/ehp.1104056 PMC3346775

[85] RStudio Team, Boston MA. RStudio: Integrated Development Environment for R [Internet]. PBC; 2022. Available from: www.rstudio.com.

[86] Lavaan RY. An R package for structural equation modeling. J Stat Softw. 2012;48:1–36. 10.18637/jss.v048.i02. 10.18637/jss.v048.i02

[87] Brown L, Sherbenou RJ, Johnson SK. Test of Nonverbal Intelligence. Third. Austin, TX: Pro-Ed; 1997.

[88] Maas CJM, Hox JJ. Sufficient sample sizes for multilevel modeling. Methodol (Gott). 2005;1(3):86–92.

[89] Schumacker RE, Lomax RG. A beginner’s guide to structural equation modeling: Fourth Edition. Routledge; 2015. p. 394.

[90] Cudeck. of Assessing Model Fit. Testing structural equation models [Internet].1993; Available from: https://books.google.com/books?hl=en&lr=&id=FvIxxeYDLx4C&oi=fnd&pg=PA136&ots=_N_DzWXGAQ&sig=x88l8Kcc8yTFFL_xW6r-HsQZ3KA

[91] Jöreskog KG, Sörbom D. LISREL 8: structural equation modeling with the SIMPLIS Command Language. Scientific Software International; 1993. p. 226.

[92] Browne MW, Cudeck R. Alternative ways of assessing model fit. Sociol Methods Res. 1992;21(2):230–58. 10.1177/0049124192021002005

[93] Little TD. Longitudinal structural equation modeling. Guilford Press; 2013. p. 386.

[94] Pavlov G, Maydeu-Olivares A, Shi D. Using the standardized root mean squared residual (SRMR) to assess exact fit in structural equation models. Educ Psychol Meas. 2021;81(1):110–30. 33456064 10.1177/0013164420926231 PMC7797960

[95] McCormick EM, Byrne ML, Flournoy JC, Mills KL, Pfeifer JH. The Hitchhiker’s guide to longitudinal models: a primer on model selection for repeated-measures methods. Dev Cogn Neurosci. 2023;63(101281):101281. 37536082 10.1016/j.dcn.2023.101281 PMC10412784

[96] DiPietro JA, Voegtline KM. The gestational foundation of sex differences in development and vulnerability. Neuroscience. 2017;342:4–20. 26232714 10.1016/j.neuroscience.2015.07.068 PMC4732938

[97] Cho S-M, Kim EJ, Lim K-Y, Lee J-W, Shin Y-M. The effects of maternal depression on child mental health problems based on gender of the child. Community Ment Health J. 2015;51(3):354–8. 25566948 10.1007/s10597-014-9824-6

[98] Saunders CR, Ramesh A, Shockley DC. Modulation of neurotoxic behavior in F-344 rats by temporal disposition of benzo(a)pyrene. Toxicol Lett. 2002;129(1–2):33–45. 11879972 10.1016/S0378-4274(01)00467-2

[99] Saunders CR, Das SK, Ramesh A, Shockley DC, Mukherjee S. Benzo(a)pyrene-induced acute neurotoxicity in the F-344 rat: role of oxidative stress. J Appl Toxicol. 2006;26(5):427–38. 16858674 10.1002/jat.1157

[100] Andersson H, Lindqvist E, Westerholm R, Grägg K, Almén J, Olson L. Neurotoxic effects of fractionated diesel exhausts following microinjections in rat hippocampus and striatum. Environ Res. 1998;76(1):41–51. 9466896 10.1006/enrs.1997.3791

[101] Jett DA, Navoa RV, Lyons MA Jr. Additive inhibitory action of chlorpyrifos and polycyclic aromatic hydrocarbons on acetylcholinesterase activity in vitro. Toxicol Lett. 1999;105(3):223–9. 10355543 10.1016/S0378-4274(99)00010-7

[102] Tzekova A, Leroux S, Viau C. Electrophilic tissue burden in male Sprague-Dawley rats following repeated exposure to binary mixtures of polycyclic aromatic hydrocarbons. Arch Toxicol. 2004;78(2):106–13. 14520510 10.1007/s00204-003-0518-z

[103] Ruhé HG, Mason NS, Schene AH. Mood is indirectly related to serotonin, norepinephrine and dopamine levels in humans: a meta-analysis of monoamine depletion studies. Mol Psychiatry. 2007;12(4):331–59. 17389902 10.1038/sj.mp.4001949

[104] Dong M-X, Chen G-H, Hu L. Dopaminergic system alteration in anxiety and compulsive disorders: a systematic review of Neuroimaging studies. Front Neurosci. 2020;14:608520. 33343291 10.3389/fnins.2020.608520 PMC7744599

[105] Lovasi GS, Treat CA, Fry D, Shah I, Clougherty JE, Berberian A, et al. Clean fleets, different streets: evaluating the effect of New York City’s clean bus program on changes to estimated ambient air pollution. J Expo Sci Environ Epidemiol. 2023;33(3):332–8. 35906405 10.1038/s41370-022-00454-5 PMC10234802

[106] Halligan SL, Murray L, Martins C, Cooper PJ. Maternal depression and psychiatric outcomes in adolescent offspring: a 13-year longitudinal study. J Affect Disord. 2007;97(1–3):145–54. 16863660 10.1016/j.jad.2006.06.010

[107] Campbell SB, Morgan-Lopez AA, Cox MJ, McLoyd VC. A latent class analysis of maternal depressive symptoms over 12 years and offspring adjustment in adolescence. J Abnorm Psychol. 2009;118(3):479–93. 19685946 10.1037/a0015923 PMC2729503

[108] Steel Z, Marnane C, Iranpour C, Chey T, Jackson JW, Patel V, et al. The global prevalence of common mental disorders: a systematic review and meta-analysis 1980–2013. Int J Epidemiol. 2014;43(2):476–93. 24648481 10.1093/ije/dyu038 PMC3997379

[109] Baxter AJ, Scott KM, Vos T, Whiteford HA. Global prevalence of anxiety disorders: a systematic review and meta-regression. Psychol Med. 2013;43(5):897–910. 22781489 10.1017/S003329171200147X

[110] Perera FP, Chang H-W, Tang D, Roen EL, Herbstman J, Margolis A, et al. Early-life exposure to polycyclic aromatic hydrocarbons and ADHD behavior problems. PLoS ONE. 2014;9(11):e111670. 25372862 10.1371/journal.pone.0111670 PMC4221082

[111] O’Donnell K, O’Connor TG, Glover V. Prenatal stress and neurodevelopment of the child: focus on the HPA axis and role of the placenta. Dev Neurosci. 2009;31(4):285–92. 19546565 10.1159/000216539

[112] McEwen BS, Tucker P. Critical biological pathways for chronic psychosocial stress and research opportunities to advance the consideration of stress in chemical risk assessment. Am J Public Health. 2011;101(Suppl 1Suppl 1):S131–9. 22021312 10.2105/AJPH.2011.300270 PMC3222511

[113] Sandman CA, Davis EP, Buss C, Glynn LM. Exposure to prenatal psychobiological stress exerts programming influences on the mother and her fetus. Neuroendocrinology. 2012;95(1):7–21. 21494029 10.1159/000327017 PMC7068789

[114] Hopson MB, Margolis A, Rauh V, Herbstman J. Impact of the home environment on the relationship between prenatal exposure to environmental tobacco smoke and child behavior. Int J Child Health Hum Dev. 2016;9(4):453–64. 28845210 PMC5570618

[115] Lovejoy MC, Graczyk PA, O’Hare E, Neuman G. Maternal depression and parenting behavior: a meta-analytic review. Clin Psychol Rev. 2000;20(5):561–92. 10860167 10.1016/S0272-7358(98)00100-7

[116] Thomas MMC. Latent classes and longitudinal patterns of material hardship as predictors of child well-being. Child Soc [Internet]. 2023; https://onlinelibrary.wiley.com/doi/10.1111/chso.12687 10.1111/chso.12687 PMC10923602 38464906

[117] Bellair PE, McNulty TL, Roscigno VJ, Lei MK. Childhood material hardship and externalizing behavior. Justice Q. 2021;38(3):454–78. 10.1080/07418825.2019.1584326

[118] Peverill M, Dirks MA, Narvaja T, Herts KL, Comer JS, McLaughlin KA. Socioeconomic status and child psychopathology in the United States: a meta-analysis of population-based studies. Clin Psychol Rev. 2021;83:101933. 33278703 10.1016/j.cpr.2020.101933 PMC7855901

