# Supplementary material for: Polycyclic aromatic hydrocarbon exposure effects on trajectories of maternal and adolescent mental health
Source: Child Adolesc Psychiatry Ment Health. 2024 Sep 11;18:114. doi: 10.1186/s13034-024-00804-1 (PMC11391764; doi:10.1186/s13034-024-00804-1)
Supplement: Supplementary file 1 — Supplementary material 1 [file 13034_2024_804_MOESM1_ESM.docx]

**Supplementary Methods**

**Maternal and Family Factors Included in Structural Equation Models**

Maternal ethnoracial identification was determined by maternal self-report. For the purposes of the current analyses, ethnoracial identification was binarized into either Black or Latiné. Maternal years of education were acquired by self-reported years of education after the first grade, obtained at the third trimester visit. Infants’ sex at birth and gestational age were determined by medical chart review at birth. Presence of a smoker in the home was self-reported by mothers during the third trimester visit. Home heat sources are known to increase indoor air pollution; as such, heating season is a derived variable to control for indoor air pollution confounding and is based on children’s date of birth (i.e., November to April) [1,2]. Maternal intelligence was operationalized as mothers’ total score on the Test of Nonverbal Intelligence, third edition (TONI-3; [3]). The TONI-3 was administered at children’s preschool visit (child mean age = 3.823, SD = 2.18).

**Supplementary Results**

*Participants*

Table S1 presents age 16 self-reported anxiety and externalizing problems scores. Figure S1 presents trajectories of maternal demoralization over time.

| Table S1. Age 16 Mental Health Self-Report Measures | | | | |
| --- | --- | --- | --- | --- |
| Scale | N | Mean (SD) | Min | Max |
| RCMAS Total Raw Score | 313 | 8.97 (5.81) | 0 | 24 |
| YSR Externalizing Problems T-Score | 281 | 49.5 (9.30) | 29 | 72 |
| Note. RCMAS=Revised Children's Manifest Anxiety Scale, YSR= Youth Self-Report | | | | |


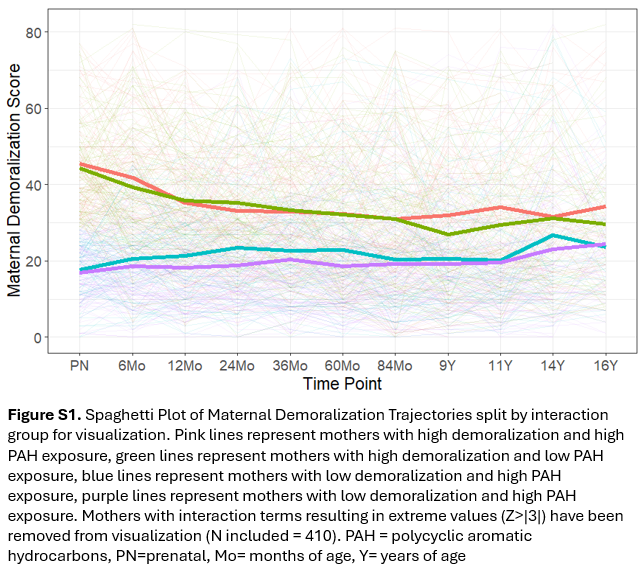


*Latent growth of Maternal Demoralization with only Mothers with 3 or more timepoints of data*

The latent growth model indicated good model fit (RMSEA = 0.07, RMSEA p-value<0.01, CFI=0.89, SRMR=0.06). Presence (v. absence) of PAH-DNA adducts was associated with higher prenatal maternal demoralization (intercept β = 3.03, SE = 1.51, *p* =0.04; Table 3) but not the trajectory of change in maternal demoralization over time (slope β = -0.01, SE = 0.01, *p* =NS; Table S2). Main effects of race (β = -4.23, SE = 2.12, *p* =0.04; Table X) and a smoker living at home (β = 3.54, SE = 0.78, *p* < 0.01; Table S2) were also significant. Latiné mothers reported higher prenatal demoralization, as did mothers living with a smoker, who also reported smaller decreases in demoralization over time (β = -0.01, SE = 0.01, *p* =0.03, Table S2). Across the entire sample, the average predicted demoralization score was approximately 39 (β=38.57; *p_intercept_*<0.01), representing moderate levels of demoralization (total score range = 0-108), which declined over time (β=-0.08; *p_slope_*=0.01). Finally, the covariance of the intercept and the slope differed from zero (β=-0.33; *p*<0.01), indicating that prenatal demoralization scores correlated with the change in demoralization over time.

Table S2. Latent Growth Curve Model Results - 3 or more time points of data

| Outcome: | Demoralization Intercept | | | Demoralization Slope | | | |
| --- | --- | --- | --- | --- | --- | --- | --- |
| *Predictor* | Coefficient | z-value | p-value | | Coefficient | z-value | p-value |
| Maternal PAH-DNA Adducts | 3.03 | 2.01 | 0.04 | | -0.01 | -0.80 | 0.42 |
| Gestational Age | -1.09 | -1.43 | 0.15 | | 0.004 | 0.74 | 0.46 |
| Maternal Age at Birth | 1.35 | 1.62 | 0.11 | | 0.01 | 1.02 | 0.31 |
| Maternal Nativity | -3.61 | -1.62 | 0.11 | | 0.02 | 1.51 | 0.13 |
| Maternal Prenatal Years of Education | -0.70 | -0.81 | 0.42 | | -0.01 | -0.85 | 0.39 |
| Smoker at Home | 3.54 | 4.51 | 0.00 | | -0.01 | -2.16 | 0.03 |
| Heat Season | 0.25 | 0.16 | 0.87 | | -0.002 | -0.20 | 0.84 |
| Maternal Intelligence | -0.44 | -0.51 | 0.61 | | 0.003 | 0.55 | 0.58 |
| Maternal Ethnoracial Identification | -4.23 | -2.00 | 0.04 | | 0.02 | 1.37 | 0.17 |

Note: PAH = polycyclic aromatic hydrocarbons

*Latent growth of Maternal Demoralization Predicting Child Anxiety Symptoms at Age 14*

The latent growth model indicated good model fit (RMSEA = 0.06, RMSEA p-value<0.01, CFI=0.9, SRMR=0.05). Latent growth modeled using lambda free parameters to estimate the slope did not significantly change model fit (RMSEA = 0.05, RMSEA p-value=NS, CFI=0.93, SRMR=0.06). Exposure to PAH-DNA adducts was associated with higher maternal demoralization at the prenatal visit (β = 3.02, SE = 1.51, *p* =0.04; growth model intercept) but not the trajectory of change in maternal demoralization over time. Main effects of race (β = -4.19, SE = 2.12, *p* =0.04) and a smoker living at home (β = 3.54, SE = 0.78, *p* < 0.01) were also significant such that Latiné mothers and mothers with living with a smoker indicated higher prenatal demoralization (intercept). Mothers living with a smoker additionally demonstrated less decrease in maternal demoralization over time (β = -0.01, SE = 0.01, *p* =0.03).

*Latent growth of Maternal Demoralization Predicting Child Anxiety Symptoms at Age 16*

The latent growth model indicated adequate model fit (RMSEA = 0.06, RMSEA p-value<0.01, CFI=0.9, SRMR=0.08). Exposure to PAH-DNA adducts were associated with higher maternal demoralization at the prenatal visit (β = 3.01, SE = 1.51, *p* = 0.04, Table S3) but not the trajectory of change in maternal demoralization over time. Main effects of race (β = -2.03, SE = 1.02, *p* =0.04) and a smoker living at home (β = 3.54, SE = 0.78, *p* < 0.01) were also significant such that Latiné mothers and mothers with living with a smoker indicated higher prenatal demoralization. Mothers living with a smoker additionally demonstrated less decrease in maternal demoralization over time (β = -0.01, SE = 0.01, *p* =0.03).

Table S3. Latent Growth Curve Model Results - Child Anxiety at Age 16

| Variable | Intercept | | | Slope | | | |
| --- | --- | --- | --- | --- | --- | --- | --- |
|  | Coefficient | z-value | p-value | | Coefficient | z-value | p-value |
| Maternal PAH-DNA Adducts | 3.01 | 2.00 | 0.04 | | -0.01 | -0.74 | 0.46 |
| Gestational Age | -1.09 | -1.44 | 0.15 | | 0.004 | 0.76 | 0.45 |
| Maternal Age at Birth | 1.35 | 1.62 | 0.11 | | 0.01 | 1.00 | 0.32 |
| Maternal Nativity | -3.62 | -1.62 | 0.11 | | 0.02 | 1.51 | 0.13 |
| Maternal Prenatal Years of Education | -0.70 | -0.81 | 0.42 | | -0.01 | -0.87 | 0.38 |
| Smoker at Home | 3.54 | 4.52 | 0.00 | | -0.01 | -2.19 | 0.03 |
| Heat Season | 0.25 | 0.17 | 0.87 | | -0.003 | -0.24 | 0.82 |
| Maternal Intelligence | -0.45 | -0.53 | 0.60 | | 0.004 | 0.62 | 0.54 |
| Maternal Ethnoracial Identification | -2.03 | -1.98 | 0.04 | | 0.01 | 1.32 | 0.19 |
| Child Anxiety | Coefficient | | z-value | | | p-value | |
| Intercept  Slope | 0.02 | | 4.95 | | | 0.00 | |
|  | 3.56 | | 3.39 | | | 0.001 | |
| Maternal Prenatal Years of Education | -0.01 | | -0.12 | | | 0.91 | |
| Child Sex at Birth | -0.35 | | -4.65 | | | 0.00 | |

Note: PAH = polycyclic aromatic hydrocarbons

*Latent growth of Maternal Demoralization Predicting Child Depression Symptoms at Age 14*

The latent growth model indicated adequate model fit (RMSEA = 0.06, RMSEA p-value<0.01, CFI=0.9, SRMR=0.05). Latent growth modeled using lambda free parameters to estimate the slope did not significantly change model fit (RMSEA = 0.05, RMSEA p-value=NS, CFI=0.92, SRMR=0.07). Exposure to PAH-DNA adducts was associated with higher maternal demoralization at the prenatal visit (β = 3.03, SE = 1.51, *p =* 0.04) but not the trajectory of change in maternal demoralization over time. Main effects of race (β = -2.05, SE = 1.02, *p* =0.04) and a smoker living at home (β = 3.53, SE = 0.78, *p* < 0.01) were also significant such that Latiné mothers and mothers with living with a smoker indicated higher prenatal demoralization. Mothers living with a smoker additionally demonstrated less decrease in maternal demoralization over time (β = -0.01, SE = 0.01, *p* =0.03).

*Latent growth of Maternal Demoralization Predicting Child Externalizing Symptoms at Age 14*

The latent growth model indicated good model fit (RMSEA = 0.06, RMSEA p-value<0.01, CFI=0.9, SRMR = 0.08). Latent growth modeled using lambda free parameters to estimate the slope did not significantly change model fit (RMSEA = 0.06, RMSEA p-value=NS, CFI=0.91, SRMR=0.07). Exposure to PAH-DNA adducts were associated with higher maternal demoralization at the prenatal visit (β = 3.03, SE = 1.44, *p* = 0.04) but not the trajectory of change in maternal demoralization over time. Main effects of race (β = -2.01, SE = 0.98, *p* =0.04) and a smoker living at home (β = 3.55, SE = 0.75, *p* < 0.01) were also significant such that Latiné mothers and mothers with living with a smoker indicated higher prenatal demoralization. Mothers living with a smoker additionally demonstrated less decrease in maternal demoralization over time (β = -0.01, SE = 0.01, *p* =0.02).

*Latent growth of Maternal Demoralization Predicting Child Externalizing Symptoms at Age 16*

The latent growth model indicated poor model fit (RMSEA = 0.06, RMSEA p-value<0.01, CFI=0.9, SRMR = 0.05). Exposure to PAH-DNA adducts were associated with higher maternal demoralization at the prenatal visit (β = 3.03, SE = 1.51, *p* = 0.04, Table S4) but not the trajectory of change in maternal demoralization over time. Main effects of race (β = -2.05, SE = 1.02, *p* =0.04) and a smoker living at home (β = 3.54, SE = 0.78, *p* < 0.01) were also significant such that Latiné mothers and mothers with living with a smoker indicated higher prenatal demoralization. Mothers living with a smoker additionally demonstrated less decrease in maternal demoralization over time (β = -0.01, SE = 0.01, *p* =0.03).

Table S4. Latent Growth Curve Model Results - Child Externalizing Problems at Age 16

| Variable | Intercept | | | Slope | | | |
| --- | --- | --- | --- | --- | --- | --- | --- |
|  | Coefficient | z-value | p-value | | Coefficient | z-value | p-value |
| Maternal PAH-DNA Adducts | 3.02 | 2.01 | 0.04 | | -0.01 | -0.81 | 0.42 |
| Gestational Age | -1.10 | -1.45 | 0.15 | | 0.004 | 0.78 | 0.43 |
| Maternal Age at Birth | 1.41 | 1.68 | 0.09 | | 0.01 | 0.88 | 0.38 |
| Maternal Nativity | -3.61 | -1.62 | 0.11 | | 0.02 | 1.52 | 0.13 |
| Maternal Prenatal Years of Education | -0.71 | -0.82 | 0.41 | | -0.01 | -0.84 | 0.40 |
| Smoker at Home | 3.54 | 4.53 | 0.00 | | -0.01 | -2.19 | 0.03 |
| Heat Season | 0.21 | 0.14 | 0.89 | | -0.002 | -0.16 | 0.87 |
| Maternal Intelligence | -0.48 | -0.56 | 0.58 | | 0.004 | 0.66 | 0.51 |
| Maternal Ethnoracial Identification | -2.05 | -2.01 | 0.04 | | 0.01 | 1.38 | 0.17 |
| Child Externalizing Problems | Coefficient | | z-value | | | p-value | |
| Intercept  Slope | 0.00 | | 0.11 | | | 0.91 | |
|  | 2.79 | | 2.39 | | | 0.02 | |
| Maternal Prenatal Years of Education | -0.01 | | -0.16 | | | 0.87 | |
| Child Sex at Birth | 0.001 | | 0.02 | | | 0.99 | |

Note: PAH = polycyclic aromatic hydrocarbons

*Latent growth of Maternal Demoralization and Maternal PAH-DNA Adducts Predicting Child Anxiety Symptoms at Age 14*

The latent growth model indicated adequate model fit (RMSEA = 0.06, RMSEA p-value<0.01, CFI=0.9, SRMR=0.08). Exposure to maternal PAH-DNA adducts was not associated with child self-reported anxiety symptoms (β = -0.10, SE = 0.12, *p* =NS).

*Latent growth of Maternal Demoralization and Maternal PAH-DNA Adducts Predicting Child Depression Symptoms at Age 14*

The latent growth model indicated adequate model fit (RMSEA = 0.06, RMSEA p-value<0.01, CFI=0.9, SRMR=0.08). Exposure to maternal PAH-DNA adducts was not associated with child self-reported anxiety symptoms (β = -0.03, SE = 0.11, *p* =NS).

*Latent growth of Maternal Demoralization and Maternal PAH-DNA Adducts Predicting Child Externalizing Symptoms at Age 14*

The latent growth model indicated good model fit (RMSEA = 0.07, RMSEA p-value<0.01, CFI=0.9, SRMR = 0.08). Exposure to maternal PAH-DNA adducts was not associated with child self-reported anxiety symptoms (β = -0.06, SE = 0.11, *p* =NS).

Table S5. Latent Growth Curve Model Results – maternal PAH exposure predicting Child Anxiety at Age 14

| Variable | Intercept | | | Slope | | | |
| --- | --- | --- | --- | --- | --- | --- | --- |
|  | Coefficient | z-value | p-value | | Coefficient | z-value | p-value |
| Maternal PAH-DNA Adducts | 2.96 | 2.05 | 0.04 | | -0.01 | -0.68 | 0.50 |
| Gestational Age | -1.07 | -1.47 | 0.14 | | 0.004 | 0.75 | 0.45 |
| Maternal Age at Birth | 1.38 | 1.73 | 0.08 | | 0.01 | 0.98 | 0.33 |
| Maternal Nativity | -3.63 | -1.70 | 0.09 | | 0.03 | 1.65 | 0.10 |
| Maternal Prenatal Years of Education | -0.74 | -0.90 | 0.37 | | -0.01 | -0.82 | 0.41 |
| Smoker at Home | 747 | 4.69 | <0.001 | | -0.03 | -2.30 | 0.02 |
| Heat Season | 0.34 | 0.23 | 0.82 | | -0.003 | -0.3 | 0.76 |
| Maternal Intelligence | -0.44 | -0.54 | 0.59 | | 0.004 | 0.63 | 0.53 |
| Maternal Ethnoracial Identification | -4.13 | -2.04 | 0.04 | | 0.02 | 1.29 | 0.20 |
| Child Anxiety | Coefficient | | z-value | | | p-value | |
| Intercept  Slope | 0.01 | | 1.55 | | | 0.12 | |
|  | 1.99 | | 2.06 | | | 0.04 | |
| Maternal Prenatal Years of Education | 0.03 | | 0.49 | | | 0.63 | |
| Child Sex at Birth | -0.51 | | -4.57 | | | <0.001 | |
| **Maternal PAH-DNA Adducts** | **0.10** | | **0.83** | | | **0.41** | |
| Smoker at Home | -0.15 | | -1.26 | | | 0.21 | |
| Heat Season | -0.02 | | -0.19 | | | 0.85 | |

Note: PAH = polycyclic aromatic hydrocarbons

Table S6. Latent Growth Curve Model Results - maternal PAH exposure predicting Child Depression Problems at Age 14

| Variable | Intercept | | | Slope | | | |
| --- | --- | --- | --- | --- | --- | --- | --- |
|  | Coefficient | z-value | p-value | | Coefficient | z-value | p-value |
| Maternal PAH-DNA Adducts | 2.99 | 2.07 | 0.04 | | -0.01 | -0.75 | 0.46 |
| Gestational Age | -1.11 | -1.53 | 0.13 | | 0.004 | 0.82 | 0.41 |
| Maternal Age at Birth | 1.38 | 1.73 | 0.09 | | 0.01 | 1.08 | 0.28 |
| Maternal Nativity | -3.60 | -1.69 | 0.09 | | 0.03 | 1.65 | 0.10 |
| Maternal Prenatal Years of Education | -0.72 | -0.87 | 0.39 | | -0.01 | -0.96 | 0.34 |
| Smoker at Home | 7.50 | 4.72 | <0.001 | | -0.03 | -2.28 | 0.02 |
| Heat Season | 0.25 | 0.18 | 0.86 | | -0.002 | -0.18 | 0.86 |
| Maternal Intelligence | -0.43 | -0.52 | 0.60 | | 0.003 | 0.57 | 0.57 |
| Maternal Ethnoracial Identification | -4.28 | -2.11 | 0.04 | | 0.02 | 1.41 | 0.16 |
| Child Depression | Coefficient | | z-value | | | p-value | |
| Intercept  Slope | 0.01 | | 1.71 | | | 0.09 | |
|  | 1.13 | | 1.09 | | | 0.27 | |
| Maternal Prenatal Years of Education | -0.04 | | -0.60 | | | 0.55 | |
| Child Sex at Birth | -0.38 | | -3.23 | | | 0.001 | |
| **Maternal PAH-DNA Adducts** | **-0.03** | | **-0.29** | | | **0.77** | |
| Smoker at Home | 0.01 | | 0.07 | | | 0.94 | |
| Heat Season | -0.01 | | -0.12 | | | 0.91 | |

Note: PAH = polycyclic aromatic hydrocarbons

Table S7. Latent Growth Curve Model Results - maternal PAH exposure predicting Child Externalizing Problems at Age 16

| Variable | Intercept | | | Slope | | | |
| --- | --- | --- | --- | --- | --- | --- | --- |
|  | Coefficient | z-value | p-value | | Coefficient | z-value | p-value |
| Maternal PAH-DNA Adducts | 3.00 | 2.08 | 0.04 | | -0.01 | -0.78 | 0.44 |
| Gestational Age | -1.06 | -1.46 | 0.14 | | 0.003 | 0.68 | 0.50 |
| Maternal Age at Birth | 1.39 | 1.73 | 0.08 | | 0.01 | 1.09 | 0.28 |
| Maternal Nativity | -3.71 | -1.74 | 0.08 | | 0.03 | 1.78 | 0.08 |
| Maternal Prenatal Years of Education | -0.72 | -0.88 | 0.38 | | -0.01 | -0.95 | 0.34 |
| Smoker at Home | 7.55 | 4.75 | <0.001 | | -0.03 | -2.34 | 0.02 |
| Heat Season | 0.28 | 0.20 | 0.85 | | -0.002 | -0.24 | 0.81 |
| Maternal Intelligence | -0.41 | -0.49 | 0.62 | | 0.003 | 0.51 | 0.61 |
| Maternal Ethnoracial Identification | -4.15 | -2.05 | 0.04 | | 0.02 | 1.26 | 0.21 |
| Child Externalizing Problems | Coefficient | | z-value | | | p-value | |
| Intercept  Slope | 0.01 | | 1.01 | | | 0.32 | |
|  | 3.59 | | 3.38 | | | 0.001 | |
| Maternal Prenatal Years of Education | 0.08 | | 1.27 | | | 0.21 | |
| Child Sex at Birth | -0.24 | | -1.95 | | | 0.05 | |
| **Maternal PAH-DNA Adducts** | **-0.06** | | **-0.55** | | | **0.59** | |
| Smoker at Home | 0.03 | | 0.23 | | | 0.82 | |
| Heat Season | 0.02 | | 0.14 | | | 0.89 | |

Note: PAH = polycyclic aromatic hydrocarbons

Supplementary References

1. Jung KH, Patel MM, Moors K, Kinney PL, Chillrud SN, Whyatt R, et al. Effects of Heating Season on Residential Indoor and Outdoor Polycyclic Aromatic Hydrocarbons, Black Carbon, and Particulate Matter in an Urban Birth Cohort. Atmos Environ . 2010;44:4545–52.

2. Gould CF, Chillrud SN, Phillips D, Perzanowski MS, Hernández D. Soot and the city: Evaluating the impacts of Clean Heat policies on indoor/outdoor air quality in New York City apartments. PLoS One. 2018;13:e0199783.

3. Brown L, Sherbenou RJ, Johnson SK. Test of Nonverbal Intelligence. third. Austin, TX: Pro-Ed; 1997.
